# Cases of the "Peculiar Species of Convulsion" Described by the Late Dr. John Clarke, Occurring Simultaneously in Twins; with Remarks

**Published:** 1831-01

**Authors:** Marshall Hall


### CONVULSIONS.

*Cases of the "Peculiar Species of Convulsion" described by the late* DR. JOHN CLARKE, *occurring simultaneously in Twins; with Remarks.* By MARSHALL HALL, M.D. F.R.S.E. &c. [In a Letter to MR. NORTH.]

DEAR SIR: These cases of the "peculiar species of convulsion" described by the late DR. JOHN CLARKE, are quoted and sketched, rather than described, in this paper, chiefly for the sake of the opportunity thus afforded me of presenting a few cursory remarks on this interesting morbid affection of infants.

The two little patients were twin brothers, aged nearly nine months. They became affected, nearly simultaneously, by restlessness during the night, and with a hooping or crowing noise in the breathing, three weeks before any symptom occurred which gave alarm.

On the 27th of February, 1824, Master F. L. G. was observed to be indisposed, it was supposed, from cold. The medical friend of the family was sent for in the evening: meantime, however, the infant had fallen asleep, and seemed composed.

On the next day, at eleven A.M., the little patient was again visited: it was perfectly lively. All on a sudden it gave a slight hoop. The gums were promptly lanced; three grains of the hydrargyri submurias were prescribed to be administered immediately, and to be repeated in four hours. In the evening, it was found that the bowels had been moved freely three or four times, and that no hooping or crowing noise had taken place during the day. It was reported, indeed, that the little patient was quite well. On examining the hand, however, it was found that the thumb was firmly drawn to the palm. In a short time, too, the crowing returned, and it gradually increased. The gums were again freely lanced, and leeches were applied to the throat, which bled sufficiently to induce a little faintness; the hydrargyri submurias was prescribed in the dose of two grains every two hours, and enemata were administered.

About two o'clock in the morning, the little patient was attacked by a violent fit of convulsion. He was put into a warm bath immediately, and cold water was dashed into the face until he was restored. I saw this little boy at four o'clock A.M.

There was no return of convulsion until the 2d of March. At eleven o'clock on that day, a fit took place, which much exceeded the former one in duration and violence.

The warm bath, the hydrargyri submurias, senna, jalap, the oleum ricini, the spiritus terebinthinæ, and enemata, with frictions, were administered. The nurse's milk was abstracted. The bowels were moved with difficulty, and not satisfactorily.

No return of fit took place until the 5th of March, on which day he had the last. At this time two incisores appeared in the under jaw. The little patient seemed to be better; but the bowels were still torpid.

Each fit was preceded by the crowing noise; but this frequently existed without being followed by a fit. The crowing was attended by a spasmodic action of the muscles situated at the upper part of the throat, and by a difficulty of inspiration. Throughout there were a clenched state of the hands and a contraction of the feet and legs. Sometimes there was difficulty in swallowing, at others not. The bladder was not freely evacuated, except by the aid of emollient clysters.

For a few days before March the 18th, this little boy appeared somewhat better. The bowels had acted, and the motions were tinged with bile. The contraction of the hands and feet was relieved.

On the 19th, he became restless, and tossed his head from side to side. He was relieved by a free evacuation of the bowels.

On the 20th, this little boy was obviously very uncomfortable, and there was again a difficulty in swallowing. The 21st was passed comfortably, and he appeared better. The legs were observed to be a little swollen. He passed a good night, was cheerful when he awoke on the morning of the 22d, but died suddenly two hours afterwards, whilst the nurse was giving him a little tea.

On examination post mortem, the general surface of the body was found pale, and the feet and legs a little swelled; but there was no exudation on making an incision into the cellular membrane. In the upper jaw there were four teeth (incisores), which pierced the alveolar processes, but were still covered by the periosteum and gums; or at least a probe pushed along the teeth under the membrane was arrested at their edge, though this might be by cicatrix, as the teeth had been many times very completely lanced. There were no teeth appearing on removing the soft parts of the under jaw, except the two which had been cut during life. The scalpel, in all other parts of the jaw, passed down to the bone.

There was nothing morbid in the external skull or dura mater; the latter was, as usual, separated with the utmost difficulty from the cranium. On raising the dura mater, the tunica arachnoidea was seen extremely distended by subjacent transparent fluid, and interspersed with large arteries and veins, which were very distinguishable by the colour of the contained blood. The tunica arachnoidea was perfectly transparent; on cutting through it, and applying a sponge to remove the subjacent fluid, four drachms by weight were collected.

On cutting into the substance of the brain, more red points appeared than usual, and altogether perhaps more fluid exuded. The ventricles contained much serum; at the least, an ounce and a half.

There were no other morbid appearances in the encephalon: no tumor, either in the dura mater or substance of the brain; no abscess.

On tracing the spinal marrow three or four inches down, no fluid or other morbid appearance was observed.

On making an incision into the thorax, all the viscera were found perfectly healthy, except the pericardium, which contained much more fluid than natural; at least, two drachms by weight. The heart was completely empty. The lungs and cavities of the pleura were free from morbid appearances.

There was certainly more redness than natural, and that from enlarged vessels, of the pharynx, epiglottis, and the rima glottidis; very marked when compared with the adjacent parts: none, however, of the trachea and œsophagus.

The viscera and cavity of the abdomen were perfectly healthy, except that the latter contained a very small portion of effused serum.

What is remarkable, the twin brother of this little patient went through a similar indisposition, at the very same time; so similar, indeed, in every respect, that I do not think it necessary to enter into any detail of his symptoms. Under similar treatment, he recovered.

I now proceed to make a few reflections upon this singular simultaneous concurrence of fits in twin brothers.

*Remarks.* 1. I would, in the first place, observe, that the cause of this affection must have been one that was common to the two infants. It might be, 1, the diet; 2, the local situation, or, 3, teething. The first causes had, however, obtained, without change, for months previously to the attack; it could scarcely, therefore, be any one of these which should operate so decidedly upon two infants at the same time, in so peculiar a manner. The only common cause, the operation of which began at the period of the attack, was teething. To this cause, then, the attack was chiefly referred in the first instance. The conjecture was subsequently confirmed by the prompt appearance of the two incisores in the lower jaw of each infant, and by appearances of dentition on the post-mortem examination of the one in whom the disease proved fatal.

2. I think it important to bear in mind that dentition may be a source of irritation, long before there is any tumor of the gum perceptible to the finger. When the gum begins to be irritated, and stretched by the advancing teeth, the injury may be propagated along the nerve to the brain.

3. It is also important to remark, that, even after full relief given to the gum by lancing, the injury may continue. Tetanus from a wound does not necessarily cease even after amputation: we have the effect to treat.

4. It is important to observe, in the next place, that one single fit induces a state of the brain which disposes to the recurrence of the fit. The nervous system does not at once recover from its state of irritation; it remains more susceptible than before.

5. It is, further, the effect of every kind of fit to induce a gorged state of the blood-vessels of the brain, similar to that observed in the countenance: this condition augments the susceptibility of the brain to further attacks of fits, and may lead to effusion. Even fits of hooping cough have this effect, and thus frequently lead to fits of other kinds, and to hydrencephalus. I have more than once known a fit of hooping cough become a fit of convulsion.

6. Not only the brain, but the heart, appears also to be morbidly affected by the muscular orgasm of a fit of convulsion. It is in this manner, I believe, that fits frequently become fatal in an instant. Diseases of the head usually induce death more slowly, as we observe in hydrencephalus. Death in apoplexy itself is not generally so immediate, as that which arises from an affection of the heart. In the case which I have briefly detailed, the last action of the heart appears to have been spasmodic contraction: not a vestige of blood was found in its cavities. This organ appears, too, to have been morbidly affected before, judging from the effusion into the pericardium.

7. Convulsions are, indeed, a multiform affection. The usual form is one which attacks the muscles of ordinary voluntary motion. The species of convulsion described by Dr. J. Clarke is only remarkable from involving a part of the respiratory system of muscles, especially those about the larynx, and those of inspiration in general. Its best designation would be *the croup-like convulsion.* Another form is that described by the late Dr. KELLIE, of Leith, as affecting the hands and feet. I have seen convulsive motions almost confined to the eye, or to some part of the face; but I never thought it worth while to give them a distinct name or epithet.

8. Convulsions are not only multiform, but complicated. Some source of irritation, some cause, is usually the first link in the chain; then there is an affection of the brain; then of some part of the muscular system; then of the heart. The convulsive action, in its turn, becomes a cause of congestion of the brain, of the heart, and possibly of some other internal organs.

9. It is also necessary to keep in view that there are other sources of convulsion, besides irritation. Teething, and a deranged condition of the alimentary canal, are certainly the most usual causes of convulsion; but another cause may be disease of the brain and spinal marrow themselves; and there is a further cause, less known, less suspected, in an exhausted condition of the system from diarrhœa or loss of blood. You, sir, will remember the peculiarly interesting case which we attended together, of hæmatemesis in an infant: convulsion occurred in the last stage of exhaustion, and carried it off.

I am, dear sir, yours very faithfully,

MARSHALL HALL.

*London; Dec.* 13, 1830.

